# Cochlear Implantation in Intralabyrinthine Schwannoma: Case Series and Systematic Review of the Literature

**DOI:** 10.3390/audiolres13020017

**Published:** 2023-02-28

**Authors:** Sebastiano Franchella, Marzia Ariano, Francesca Bevilacqua, Stefano Concheri, Elisabetta Zanoletti

**Affiliations:** Department of Neurosciences, Section of Otorhinolaryngology—Head and Neck Surgery, University of Padova, 35122 Padova, Italy

**Keywords:** cochlear implant, intralabyrinthine schwannoma, hearing outcomes

## Abstract

Intralabyrinthine schwannomas (ILS) are rare benign tumours arising from the peripheral branches of the cochlear or vestibular nerves in the membranous labyrinth, intracochlear schwannomas being the most frequent ones. When hearing is no longer feasible on the affected side, surgical removal along with simultaneous cochlear implantation can be proposed to the patient. We hereby present a systematic review of the literature on the topic, as well as two original cases from our centre (Ospedale Università degli Studi di Padova). Cochlear implantation in intracochlear schwannomas is feasible, with overall satisfactory hearing outcomes in accordance with the evidence found in the literature.

## 1. Introduction

Vestibular schwannomas are benign tumours of the schwan cells of the eighth cranial nerve: the overall incidence in the general population ranges between 1.7 and 4.2 cases per 100,000 [1]. A subgroup of the former consists of intralabyrinthine schwannomas (ILS), which arise from the peripheral branches of the cochlear or vestibular nerves, and are located in the membranous labyrinth. The incidence of this type of schwannoma was reported between 0.81 and 1.1 cases per 100,000 even if it is probably underestimated, and it has the potential to grow in the future due to a more accurate radiological diagnosis and an increased awareness of the general population about unilateral sensorineural hearing loss [1]. There are seven subgroups of ILS based on their anatomical site. Although still limited, experience on cochlear implantation in vestibular schwannoma has grown in recent years [2] and intracochlear schwannoma particularly represents a challenge.

This review focuses on cochlear implant (CI) positioning in intracochlear ILS, which is the most frequent among ILS tumours. The most common presentation of the ILS is unilateral sensorineural hearing loss, which can be sudden or progressive [3].

Single side deafness (SSD) rehabilitation via CI is proposed in order to reduce the difficulties related to sound localisation and speech perception in noise [4]. It is more challenging than for bilateral deafness, mainly because patients will continue using the natural hearing, as provided by the non-implanted ear, and because of the difficulties of postoperative rehabilitation. Nonetheless, in recent years, technological development has partially overcome this difficulty, thanks to the possibility of the direct connection of the implant to external devices via bluetooth, making it possible to electrically stimulate and rehabilitate the implanted ear without any interference to the contralateral normal side [5]. On the other hand, considering patients’ will in SSD is mandatory and of utmost importance.

We hereby present a review on the literature regarding cochlear implantation in intracochlear schwannomas, with a particular focus on audiological outcomes, and present two original cases from our experience in cochlear implantation and vestibular schwannoma management [2,6,7].

## 2. Materials and Methods

We preliminarily defined our study using the PICO protocol (Patient: patients with intracochlear vestibular schwannoma, Intervention: cochlear implantation, Outcome: hearing restoration).

**Figure 1:**
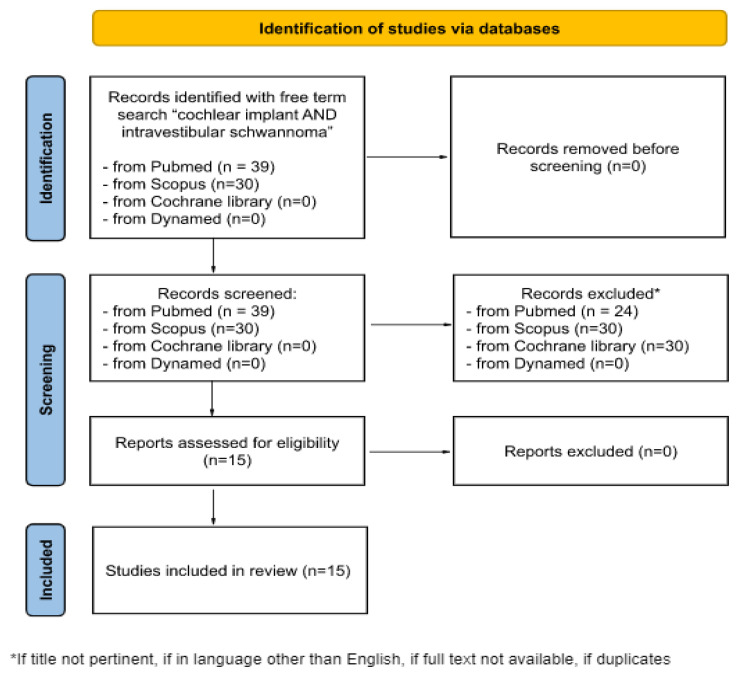
PRISMA flow diagram for the literature research on Pubmed, Scopus, Cochrane Library and Dynamed, up to December 2022.

The databases Pubmed, Scopus, Cochrane Library and Dynamed were systematically screened up to January 2023 using the free term search: “cochlear implant AND intracochlear schwannoma”. No temporal filter was set. We decided to exclude articles which were duplicates, written in a language other than English or did not have the full text available. In the end, 15 articles were included in our review (Figure 1).

The two reported original cases were managed and operated on at our tertiary reference centre: Otolaryngology, Otoneurological and skull base surgery section, University Hospital of Padova. Fully informed consent was retrieved for each patient’s clinical data, according to the declaration of Helsinki.

## 3. Results

Results are summarised in Table 1. Table 1 aimed to find out which common aspects were reported in the selected studies and assess whether cochlear implant (CI) was feasible and effective in hearing rehabilitation in intralabyrinthine schwannomas (ILS). There is a great heterogeneity of the data in the literature. The table contains general information about each study’s population, objective parameters such as preoperative and postoperative PTA (Pure Tone Average) and WRS (Word Recognition Score), the different techniques of outcome assessment, and the follow-up period. PTA was calculated in most articles on the 500−1000−2000−4000 Hz frequencies, although some works, like that of Bento et al. (2016) [8], Pastor Gomis et al. (2021) [9], De Paula Vernetta et al. (2017) [10], Patel et al. (2022) [11] and Totten et al. (2021) [12] calculated it differently. Details on the surgical technical and intraoperative pitfalls were reported when present in the studies, taking into consideration that, when talking about cochleoectomy, the general intended meaning is a surgical demolition of different parts of the cochlea, while a cohleostomy is a simple access to the hearing organ via a surgical stoma.

Sudhoff et al. (2021) [13], in their retrospective study, included seven patients with intralabyrinthine schwannoma, each of them with cochlear involvement. They stated that in their cohort, surgical approach was individually chosen according to the tumour extent. This led to the deployment of different surgical techniques, as follows. They described the push/pull technique with a test electrode to mobilise the tumour from its post-modiolar attachment and extract it. They also described the Gelfoam pusher technique; to remove the post-modiolar part of the tumour, a tight roll of dry Gelfoam was introduced into the basal turn and pushed into the scala until the residual tumour was visible and removable. As for the other patients included in this article, a drill-out technique was performed with different cochleostomy extension, depending on the tumour localisation: a two-cavity technique with a decapping of the bone from the scalar turns and subsequent repair with fascia, cartilage and bone dust after CI electrode placement, an access to the cochlea combined with a labyrinthectomy or with access to the second turn and, in one case, even the partial removal of the tumour through the basal and second turn of the cochlea. The authors reported that all the surgical techniques mentioned above had a satisfactory outcome in terms of tumour removal. All patients underwent synchronous cochlear implantation. In this article, Sudhoff et al. (2021) [13] also stated the importance of an adequate MRI (Magnetic Resonance Imaging) follow-up to exclude residual tumours after surgery. MRI assessment of ILS removal is a tool for surgery quality control [13]. The problem of CI artifacts, impeding a correct radiological visualisation of the inner ear, could be overcome by a proper implant and patient position. Moreover, the choice of the right MRI sequence is important; for example, sequences based on a MARS (material artifact reduction sequences) protocol might be used to allow a proper visualisation of the inner ear and internal auditory canal. The explanation of the magnet is theoretically possible, but as it is related to the risk of infection, it has to be proposed as a last resort [13].

Bento et al. (2016) [8], in their case report, showed a successful hearing rehabilitation with cochlear implant in a patient with bilateral hearing loss and vertigo, in a left intracochlear schwannoma. In their article, they described in great detail the surgical technique used, starting from a mastoidectomy and posterior tympanotomy, then proceeding onto the incus and tensor tympani muscle removal to expose the cochlear apex, which was then gently drilled to perform an apical cochleostomy. The tumour was then pulled out with a delicate hook and completely resected, with minimal damage to other inner ear structures, allowing a successful cochlear implantation and hearing restoration, as demonstrated by postoperative PTA (calculated on 500−1000−2000 Hz) and WRS scores [8].

In their case series of three patients (two intracochlear schwannoma and one intravestibular), Pastor Gomis et al. (2021) [9] reported their experience in CI following tumour excision with good hearing outcomes. The surgical techniques described for tumour removal were the standard cochlear implantation approach for patient one and two, with a round window opening and removal of the tumour through this route. Instead, in patient three, a labyrinthectomy was performed due to the position of the tumour. The authors did not report any surgical pitfall or difficulty, and in every patient, a single stage CI was positioned. The postoperative outcome was the intelligibility score in speech audiometry as well as postoperative PTA, calculated on 250−500−1000−2000−4000 Hz. Seriated MRI imaging was also performed to monitor the patients in their follow-up [9]. First of all, they performed a radiological surveillance one-year post-implantation and then, if there was no evidence of growth, MRI scans were performed every two years. To avoid radiological CI artifacts in the inner ear region, the authors underlined the importance of placing the implant stimulator receiver in an exaggerated posterosuperior position [9].

Häussler et al. (2021) [14] reported the experience of 10 cases who underwent surgery and cochlear implantation for intracochlear schwannomas. Compared to the other works included in this review, they stress the importance of a preoperative assessment of vestibular function for patients with preoperative vestibular disorder, performing a preoperative bedside head impulse test, caloric testing and videonystagmography. Also, all patients underwent preoperative auditory brainstem responses. The surgical approach used depended on the location of the ILS, particularly on the affected turn of the cochlea, on the affected side of the modiolus (anterior or posterior), on the location in the vestibule or semicircular canal, and lastly, on the size of the tumour. Based on these parameters, the patients underwent either an extended cochleostomy approach with posterior tympanostomy (consider an extended cochleostomy as an extended round window approach combined with the resectioning of the promontory/cochlear wall parts) or, if there was a middle or basal to apical cochlear turn tumour involvement, a subtotal cochleoectomy approach was used (defined as the removal of the promontory and parts of the cochlea). In all cases, the resected parts of the cochlea were reconstructed with fascia, temporal muscle, cartilage and fibrin glue to prevent postoperative perilymph fistula and the future dislocation of the CI electrode. For patients with an intravestibular tumour localisation, a labyrintectomy was performed. The surgical procedure described by the authors were successful in all patients but two, considering also that the two patients were lost to follow-up, and one patient opted out from using his CI. Regarding follow-up, the authors opted for a protocol including seriated Freiburg Monosyllabic Tests (FMT) performed in a sound booth at 65 dB sound pressure level (SPL). They also cite seriated PTA in their materials and methods, but the results were not available in the article. Curiously, there is no mention of a radiological follow-up [14].

Carlson et al. (2016) [15] add to the existing literature their case series of 10 patients, seven of them with a diagnosis of neurofibromatosis type 2 (even if they do not describe if the decisional process in choosing the type of surgery and implantation in these cases varied from the non-affected ones, stating that “Results from the NF2 literature support good outcomes when the cochlear nerve can be anatomically preserved and duration of deafness is not extensive”). The authors reported to have voluntarily left intracochlear tumours in situ without even attempting a tumour removal in the hope to preserve cochlear anatomy and thus maximise the possibility of a good hearing outcome. No difficulty was described in inserting the electrode through the tumour except in one patient, in which a tip fold-over occurred and a stiffer electrode with late stylet release was used to facilitate its insertion through the soft tumour. Hearing outcomes were monitored using pre and postoperative PTA and WRS and PTA (even if they do not specify on which frequencies it was calculated), as well as with an open-set word recognition score (see Table 1) [15]. For the follow-up, they opted for MRI seriated monitoring, according to the protocol described by Carlson et al. (2015), specifically for CI users [23]. Their postoperative clinical follow-up was 21 months, and thus, this work does not present data about a long-term outcome about the patients in which the ILS was left in situ [15].

In their work, Plontke et al. (2020) [16] reported a refinement in the surgical technique to approach intracochlear tumours: a subtotal cochleoectomy with ‘‘cartilage-in-perichondrium-bed’’ defect reconstruction. The postoperative hearing outcome is described via a WRS.

The same main author, in a paper dating back to 2018, presented a case series of 10 patients with good hearing outcomes after cochlear tumour removal via cochleostomy [17]. In two patients, an additional labyrinthectomy was necessary due to an additional tumour location in the vestibule, while one patient underwent a “pull-through-technique” for tumour removal. From this cohort, eight patients received a single stage cochlear implantation, while the other two opted for the insertion of a dummy electrode carrier [17]. Good hearing results at the WRS at three months follow-up were obtained in all but one patient, but in the paper, there is no mention of postoperative PTA. The authors did not use seriate follow-up MRI imaging, except in the one patient who had refused cochlear implantation [17].

De Paula Vernetta et al. (2017) [10] described the case of a patient with a successful CI implantation after the removal of an intracochlear schwannoma, with good audiological outcomes (PTA of 25 dB SPL from 250 to 4000 Hz and 90% discrimination of syllables in open lists in silence at 65 dB SPL). Computed Tomography imaging was used as a follow-up radiological technique instead of MRI [10].

Kronenberg et al. (1999) [18] reported a complete removal of the tumour through a posterior tympanotomy approach with second stage surgery for CI, with a good hearing outcome, as shown in Table 1 [18].

Ma et al. (2020) [19] reported a case of a patient diagnosed with intracochlear schwannoma, who underwent surgical removal of the mass and cochlear implantation. Regarding the surgical technique described, they opted for a transcanal endoscopic approach with elevation of a tympanomeatal flap and the removal of the handle of the malleus to better expose the promontory. The drilling of the promontory to expose the lateral basal and second turns of the cochlea was performed to allow delicate tumour extirpation with suction and gentle dissection. Subsequent CI array insertion was performed through a routinary mastoidectomy. In this article, the authors focused on the good hearing outcome obtained, although no postoperative PTA or WRS are available [19].

Patel et al. (2022) [11] presented a case series of two patients receiving endoscopic assisted surgery and cochlear implantation, for intracochlear schwannoma. Patient one presented an intravestibulocochlear schwannoma, which was excised through a translabyrinthine approach: first a mastoidectomy with facial recess approach was performed, with a following labyrinthectomy, opening the horizontal, posterior and superior semicircular canal, as well as the vestibule. Then, via the facial recess, an extended round window cochleostomy was performed, extracting the remaining tumour in the basal turn of the cochlea. Finally, through that same cochleostomy, the CI electrode was inserted, closing the defect with small pieces of temporalis muscle graft. On the other hand, Patient two presented only with an intracochlear schwannoma, so a different surgical technique was chosen. First of all, the authors performed a blind sac closure of the external auditory canal, and then, to improve the endoscopic visualisation of the promontory, they performed a canaloplasty and a removal of the skin in the medial ear canal of the tympanic membrane, malleus and incus. Then an intact canal wall mastoidectomy and facial recess approach was performed, with the drilling of an extended round window cochleostomy. A second, separate cochleostomy was then performed along the basal turn toward the ascending limb, opening the middle turn of the cochlea: this resulted in nearly a subtotal cochleoectomy. In the end, the tumour was completely excised and a CI electrode was then placed via the round window. The openings into the cochlea were packed with periosteum, a temporalis fascia graft and a muscle graft, with a final layer of fibrin tissue sealant. The middle ear and mastoid cavity were obliterated with abdominal fat [11]. As described by Patel et al. (2022) [11], endoscopy, applied to this kind of surgery, could be a useful tool to maximise the clearance of disease. Simultaneous cochlear implantation, as shown in the hearing outcomes reported in Table 1, was satisfactory in both patients. One should note that the postoperative PTA values in this article are calculated from 250 to 6000 Hz [11].

Aschendorff et al. (2017) [20] described eight patients in their retrospective study. Although no specific data about preoperative or postoperative hearing parameters are included, they stated that patients with simultaneous CI positioning show a tendency to more favourable rehabilitation results compared to those receiving CI in a second stage surgery. Moreover, the speech comprehension obtained in their cohort of implanted patients was declared comparable to that of other patients with single-sided deafness of different etiologies [20].

Plontke et al.’s (2017) case series [21] described 12 patients affected by ILS with different locations: six with intracochlear tumours, three with intravestibular tumours, one with multilocular tumour, one with transmodiolar tumour with extension to the cerebellopontine angle and another one with transotic tumour with cerebellopontine angle’s involvement. The surgical approach and subsequent cochlear implantation were chosen according to the tumour location and dimension, the need to have seriate MRI follow-up for fear of tumour recurrence and lastly on the patients’ wishes. In three patients, a labyrinthectomy and cochlear implantation were performed. One patient underwent tumour resection via posterior tympanostomy, extended cochleostomy and cochlear implantation as a single-stage procedure. Two patients had partial/subtotal cochleoectomy with a partial reconstruction of the cochlea; one of them had the insertion of a dummy electrode carrier, while the other underwent a cochlear implantation in a single-stage surgery. In two patients, translabyrinthine/transotic tumour resection was deemed necessary due to the tumour extension from the tympanic membrane or from the inner ear to the cerebellopontine angle. The patients’ cochlear (WRS, PTA from 500 to 4000 Hz) and vestibular parameters (video Head Impulse Test gain, calorics, oVEMPS, cVEMPS) were assessed at baseline and after cochlear implantation, if possible. As one can see, the postoperative hearing outcomes described in Table 1 appeared to be satisfactory (except for patient 11) [21].

Plontke at al. (2020) [22] report their surgical experience and audiological outcome using a perimodiolar malleable cochlear implant electrode array for hearing rehabilitation after subtotal cochleoectomy for ILS. Their outcome is reported to be similar to other commercially available types of perimodiolar electrode arrays, according to postoperative values of WRS and PTA from 500 to 4000 Hz [22].

Totten et al. (2021) [12] brought the most numerous cochort, with a total of 20 patients (six of them did not present with intracochlear but with intravestibular schwannoma). They proposed different methods to approach an ILS, based on the symptoms reported by the patients, and the possibility of safe surgical removal and subsequent rehabilitation. For severe disequilibrium, before resorting to tumour excision via a translabyrinthine approach (as performed in one out of 20 patients), one may consider vestibular therapy (which was successful in four out of 12 patients with vestibular symptoms). For hearing loss, it must be treated on a case-by-case basis according to the degree of hearing loss and the patients’ wishes: through hearing aids or BiCROS (as proposed to seven patients), or cochlear implantation (proposed to three patients). Surgical excision may be considered if severe hearing loss is present, and concurrent cochlear implantation is desired. In this article, two out of 20 patients received surgery for cochlear implantation without the need for tumour removal, since there was not an intracochlear involvement. In Table 1, only the results of the two out of 20 patients who underwent CI, PTA scores were calculated on 500−1000−2000−3000 Hz [12].

## 4. Cases Presentation

Our series of intralabyrinthine schwannoma involves 10 cases: eight coming from our previous published work by Zanoletti et al. [6] and two from our most recent experience, hereby described, in which CI was simultaneously placed after tumour resection in two cases.

### 4.1. Case 1

**Figure 2:**
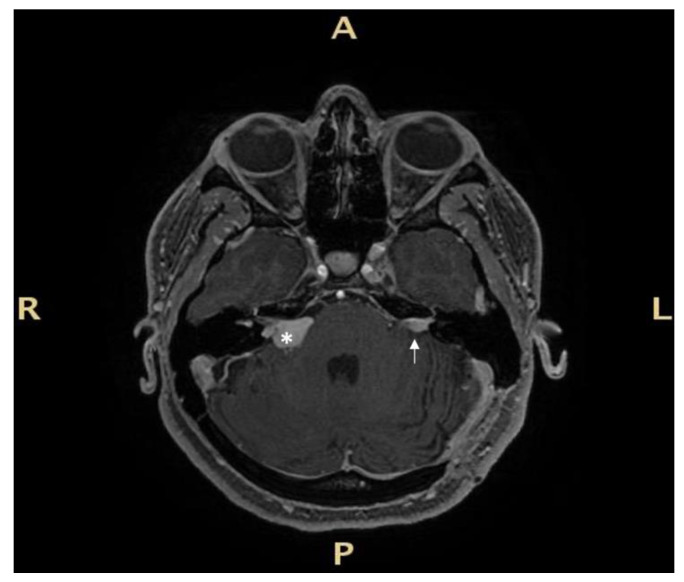
Magnetic resonance Imaging (MRI) before right cochlear implantation: right irradiated and stable tumour with intralabyrinthine component (white asterisk), and left side intrameatal recurrence (white arrow) after surgery via a retrosigmoid approach. R: right, L: left, A: anterior, P: posterior.

The first case involves a 33-year-old female patient with NF2 (Figure 2).

**Figure 3:**
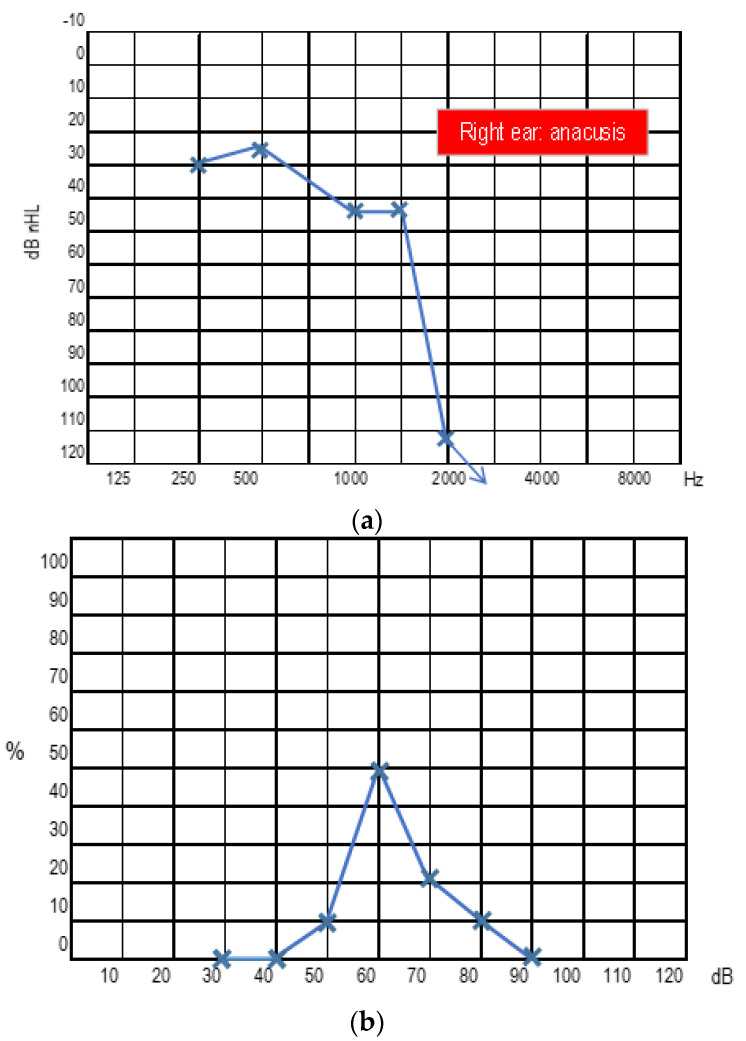
Left ear: pure tone audiometry (**a**) and speech audiometry (**b**) at preoperative evaluation. Right ear: anacusis.

In 2009, the patient underwent stereotactic radiotherapy for right eight cranial nerve schwannoma. In 2014, the patient underwent successful hearing preservation surgery via retrosigmoid approach for a left eight cranial nerve schwannoma. Hearing was preserved in the right ear for the first six years, but after 2017, it progressively diminished, ending in deafness in 2019. While right hearing was diminishing, a tumour recurrence occurred in the left side (Figure 2). Post-radiation right ear anacusis occurred, despite tumour stability, and an homolateral intracochlear mass was evidenced, while left hearing was progressively compromised due to the recurrence, as shown in Figure 3. Before treating the left only-hearing ear recurrence (where residual and not serviceable hearing was present) in 2020, she was offered the possibility of intracochlear tumour removal and cochlear implant (CI) placement.

The patient underwent right subtotal petrosectomy and EAC (external auditory canal) cul-de-sac closure for intravestibular-intracochlear schwannoma removal with simultaneous cochlear implant branded Medel, MI1200 with Flex 28 electrode. The choice of a flexible electrode was made because the objective of our surgery was a complete removal of the tumour, and after this step, the introduction of a soft array would have been possible. The tumour was inside the vestibule and in the basal turn of the cochlea, and it was completely removed before placing CI. As predicted, complete insertion of the electrode was attained, with overall good impedances. A cul-de-sac closure of the EAC was performed as planned in the subtotal petrosectomy.

One month after cochlear implant activation, the hearing outcome was 52.5 Pure Tone Average (PTA) with replicable responses at 40−45−40−45−80 dB nHL.

After three months, the hearing threshold was identifiable at 45−35−40−40−55 dB nHL (PTA 42,5), with a verbal detection at 40 dB nHL and 50% intelligibility at 60 dB. It gradually improved over the first year.

In 2021, the patient underwent surgery on the left growing recurrent schwannoma, with a translabyrinthine approach and simultaneous placement of a cochlear implant branded Medel, MI1200, with electrode array type Flex 28 mm; during surgery there was an incomplete insertion of the electrode, with two basal electrodes which remained out of the round window. Although intraoperatively good impedances were reported on all the inserted electrodes, during follow-ups, the left side CI did not provide the same benefit as the right CI, and never elicited any useful response.

**Figure 4:**
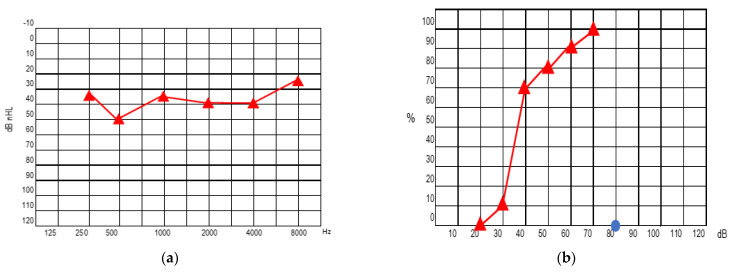
Right ear with CI: pure tone audiometry (**a**) and speech audiometry (**b**) at the last follow-up.

At the last follow-up visit, the hearing threshold with the right IC was 35−50−35−40−40−25 dB nHL (PTA 41,25) at 250−500−1000−2000−4000−6000 Hz, respectively. Vocal prosthetic audiometry (with right CI) results in 30 dB nHL verbal detection and 90% verbal intelligibility at 70 dB nHL (Figure 4). The speech evaluation showed 100% identification of vowels, 56% of consonants, and 70% recognition of sentences and 80% trisyllabic words. The patient is currently able to speak on the phone.

On the left ear there is no auditory gain, except for an improvement of tinnitus since CI placement.

### 4.2. Case 2

The second case concerns a 65-year-old man with an onset of left sensorineural hearing loss and tinnitus from 2018. At the beginning, occupational exposure to noise was considered as the aetiology of his hearing loss, as it was a symmetrical loss. The patient was invited to use hearing aids on the left side, with scarce benefits. He also underwent computed tomography (CT) and Magnetic Resonance Imaging (MRI) scans in 2020, but the radiologist only reported a possible cochlear inflammation.

**Figure 5:**
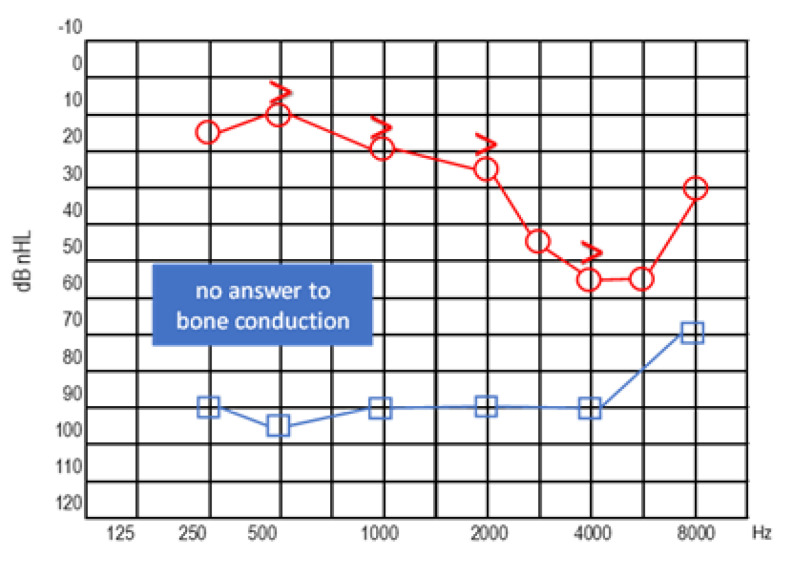
Pure tone audiometry at preoperative evaluation.

In the following years, a worsening of his left hearing was progressively observed, so the placement of a left cochlear implant (CI) was recommended to him; a device branded AB, type HiRes Ultra 3D, with slim electrode was chosen. Preoperative pure-tone audiometry shows bilateral sensorineural hearing loss, which is worse on the left as there is only mild hearing loss on the right (Figure 5).

During surgery in September 2022, the surgeon complained that the array could not be fully inserted and, after a small anterior drilling of the round window, a mass occupying the basal gyrus of the cochlea was found. Therefore, a biopsy of the neoformation and impermanent closure of the cochleostomy was carried out; the CI was temporarily housed in the postero-superior region of the temporal bone, envisaging a future positioning.

Histologically, the biopsy sample of the neoformation turned out to be a schwannoma.

In October 2022, the patient returned to the operating room for the exeresis of the left intracochlear neurinoma via subtotal petrosectomy; the drill out of the first 3–4 mm of the basal turn of the cochlea was necessary to achieve the complete removal of the neoplasia. The cochlear implant electrode was moved from its temporary site and correctly placed in the scala tympani; postoperative clinical course was uneventful. In this case, we used a soft array to avoid having to throw away an already open and fully functional cochlear implant. In addition, once the neurinoma was removed, the placement of the array had no problems.

A few days later, at first postoperative evaluation, the CI was activated; pure-tone audiometry performed with CI was 50−40−40−40−50−60 dB nhl (Pure Tone Average, PTA = 42.5) at 250−500−1000−2000−4000−6000 Hz. Speech prosthetic audiometry with CI showed a verbal detection at 40 dB nhl.

**Figure 6:**
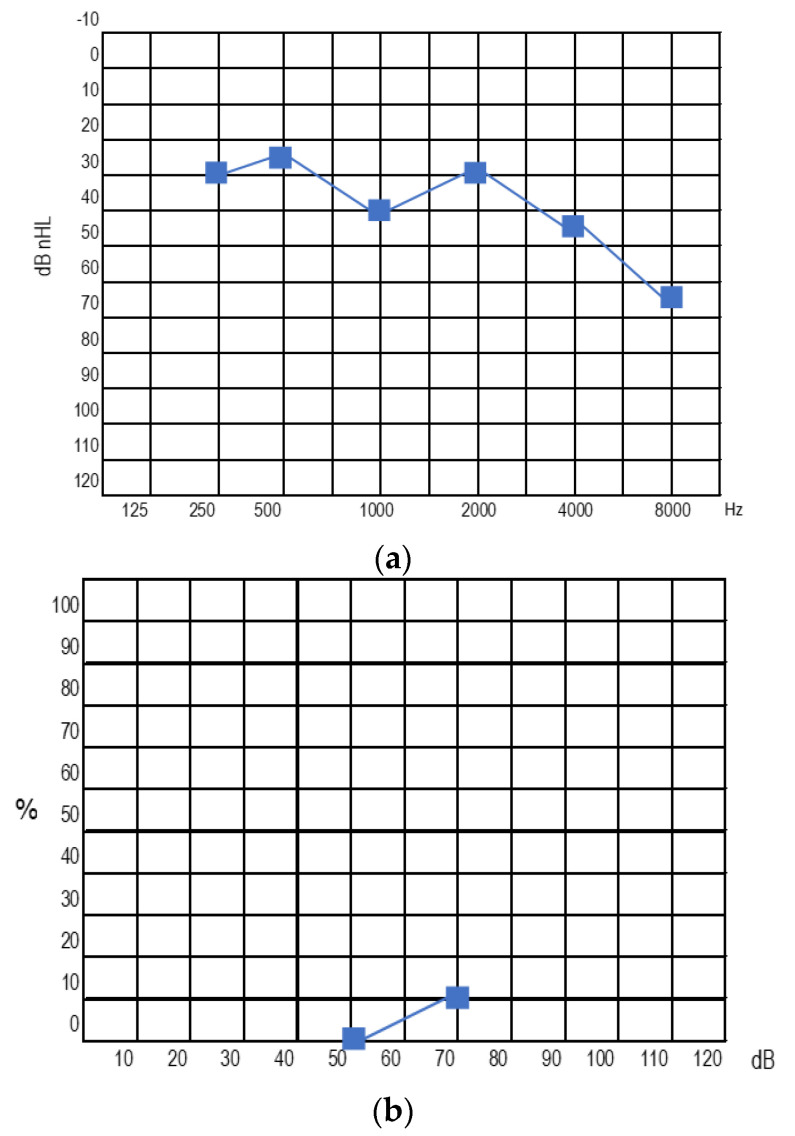
Pure tone audiometry (**a**) and speech audiometry (**b**) at last visit with left cochlear implant.

At the last visit, the hearing threshold with the left CI was 30−25−40−30−45−65 dB nHL (PTA = 35) at 250−500−1000−2000−4000−6000 Hz, respectively. Aided speech audiometry (with left CI) results in 50 dB nHL verbal detection and 10% verbal intelligibility at 70 dB nHL (Figure 6).

## 5. Discussion

Intralabyrinthine schwannomas (ILS) are rare benign tumours arising from the peripheral branches of the cochlear or vestibular nerve, and are located in the membranous labyrinth. Although their classification depends on their anatomical location, in the literature there are several. Some authors classify ILS in seven subtypes: intravestibular, intracochlear, intravestibulocochlear, transmodiolar, transmacular, transotic and tympanolabyrinthine [9,24]. Salzman et al. (2012) instead classify ILS in six subtypes: intracochlear, transmodiolar, intravestibular, vestibulocochlear, transmacular and transotic [25]. Regardless of the classification used, intracochlear and intravestibular ones are the most common, while the least common are intravestibulocochlear and transotic ones.

This review was focused on cochlear implant (CI) positioning in intracochlear ILS, which aside from being the most frequent, is also the most surgically challenging because of tumour position [13]. The placement of a CI in case of an intracochlear ILS is extremely demanding for the surgeon, who has to deal with the delicate removal of the tumour as well as the preservation of the fine structures of the inner ear before electrode insertion; the modiolus is the anatomical part at greater risk of residual microscopic tumour cells. The event of a slow ILS recurrence, especially in younger patients, has to be considered [17].

Different strategies were proposed in relation to surgical techniques for ILS excision. The “standard” cochleostomy approach as performed in our patients, is, in our opinion, the most feasible approach, but the surgeon must be aware that the drilling of the lateral wall of the cochlea makes it more difficult to keep the IC in its correct position, with consequences on its performance. Another approach is a “double cochleostomy” with “pull-through” or “push-through” of the tumour, as described by Plontke et al. [22] One limit of this technique is that it might result in incomplete tumour removal and an increased risk of inner ear structure damage due to an insufficient surgical overview [22]. For intravestibular-intracochlear tumours, the opening of the vestibule with progressive drilling towards the round window without opening it might be sufficient to remove a small tumour in the basal turn.

As for ILS’s symptoms, fluctuating, progressive or sudden hearing loss is often the early symptom at the onset. The latter is typical for intracochlear and intravestibular ILS. The mechanisms causing hearing loss are most probably related to the direct compression of the cochlear nerve, damage to the organ of Corti, and damage to the cochlear vascularisation, which ends with a reduction of the number of hair cells and spiral ganglion neurons [14]. In addition to hearing loss, several patients report tinnitus and episodes of vertigo, the latter probably caused by compression leading to endolymphatic hydrops [9,11].

ILS are currently diagnosed with Magnetic Resonance Imaging (MRI), which is able to detect and locate ILS as little as 2mm, greatly reducing the intraoperative event of ILS, which may still happen in smaller tumours as an unexpected finding in cochlear implant surgery. Their radiological appearance is of well-delimited lesions with an intermediate signal on T1-weighted imaging, while on T2-weighted imaging, an effacement of the normal intracochlear fluid can be noted; the intense and homogeneous contrast uptake in gadolinium-enhanced T1 sequences allows us to differentiate the lesion from labyrinthine haemorrhage, infectious labyrinthitis and labyrinthine ossification [9].

Treatment involves a wait-and-see attitude and surgical removal. MRI accuracy allows detection of a potential growth over time, which may lead to increasing obstruction of the cochlea, resulting in problems in CI effectiveness [21]. Surgery with or without complete tumour removal is feasible; if the tumour is partially left in situ, a portion of the cochlea must be freed to insert the electrode. Furthermore, the MRI follow-up of the tumour is technically impaired due to the magnet-related artefacts which interfere with the radiologic monitoring of tumour growth and with the detection of recurrence [19].

CI can be positioned either simultaneously or in a second stage surgery [9]. Staged cochlear implantation allows for a period of observation after surgery, and monitoring any signs of tumour recurrence thanks to effective MRI, without artefact. According to some authors, this option has to be considered in those ILS whose anatomical position makes it difficult for the surgeon to completely excise the mass, namely the multilocular, vestibulocochlear, transmodiolar, transmacular or transotic schwannomas [14]. On the other hand, two aspects have to be considered. First, there is a concrete risk of cochlear fibrosis and ossification, preventing further electrode placement in a second stage surgery. To overcome this difficulty, it is recommended to place a “dummy” electrode in the cochlea after the initial tumour resection, which acts as a placeholder for future cochlear implantation, while still allowing an adequate MRI surveillance [11,14]. Secondly, a long-time follow-up is necessary to assess tumour recurrence and for CI risks to be consequently postponed.

Regarding the preferred surgical approach, when a simultaneous tumour removal and CI positioning is planned, some authors suggest to adopt surgical refinements in the attempt to preserve a functioning cochlea, like an infero-lateral cochleostomy to open the basal turn without disrupting the round window, or partial drilling with partial opening of the cochlea turns to protect the modiolus area and the basal membrane. [8,13] On the contrary, other authors stress the feasibility of a cochlear implant regardless of the surgical approach chosen, as long as there is the preservation of key structures like the modiolus and spiral ganglion cells. [19] Placement in the vestibular scale is also feasible.

The treatment choice depends on the tumour size, the growth pattern, the preoperative ipsilateral and contralateral hearing, the possible coexistence of other symptoms, as well as general considerations like a patient’s age and general performance status [9]. Stereotactic radiosurgery is a feasible alternative, but one must bear in mind the risk of documented side effects such as deafness and damage to the facial nerve and its unclear contribution to stop the slight growth tendency of tumours such as the ILS. In fact, when radiation is administered at therapeutic dose on the cochlea, it risks encompassing the tolerated limit, and it may damage the delicate neuronal structures, especially the cochlea spiral ganglion cells. This can worsen the outcome of a cochlear implantation [20,21]. Radiotherapy is usually recommended in selected aged patients with growing tumours [21], with progressively worsening, yet still functional, hearing [12].

The limitation of this study involves first of all the great heterogeneity of the considered data of each study, as shown in Table 1. The attempt to summarise and compare the overall hearing outcomes is challenging, due to the different settings of each study, where intracochlear schwannomas were not always distinguished by intravestibular schwannoma, and the different parameters considered to assess the preoperative and postoperative hearing. PTA (Pure Tone Average) and WRS (Word Recognition Score) were the most frequently reported, but not all works included them. Duration of the follow up period of each study was also variable, with a possible confounding effect in those cases where the tumour was not removed and required a longer period of MRI surveillance, to assess recurrence, leaving aside the technical difficulties of MRI monitoring when CI was placed. No data regarding an ideal follow-up protocol could be extracted, even if most studies agreed on the need to perform seriate post-surgical MRI to monitor tumour recurrence or growth in left in situ tumours. We also must consider that none of the studies of our systematic review is at the top of the evidence pyramid, mostly because there are a large number of case reports, given the rarity of the intracochlear schwannoma.

## 6. Conclusions

Intracochlear schwannomas are rare benign tumours arising from the peripheral branches of the cochlear or vestibular nerves in the membranous labyrinth. Despite early symptoms and the possibility of early Magnetic Resonance Imaging (MRI) diagnosis, no treatment can preserve hearing. Surgery comes with complete deafness, as does the wait-and-scan attitude, even if the latter has more unpredictable hearing loss, which occurs over a long period of time. Cochlear Implant (CI) has been proposed as a rehabilitative tool both when the tumour is removed or left in site, simultaneously to tumour excision or in a second staged surgery. This review aimed to investigate the topic to explore the efficacy of CI rehabilitation, the methods of measurements in single side-deafness rehabilitation, the criteria to choose CI placement with or without tumour removal, the surgical refinements which are necessary to prevent cochlea damage and the problem of MRI surveillance for recurrence when CI is in situ. Both in the literature and our personal experience hereby reported, the hearing outcomes are promising, although the heterogeneous data and the low level of evidence did not contribute to assessing any consistent conclusions.

## Figures and Tables

**Table 1:** Summary of the studies included in our review (all data concern the implanted ear).

First Author (Year)	Design	Patients (No.)	Cochlear Involvement of the ILS	NF 2 pz	Preoperative PTA (dB)	Preoperative WRS (%)	Postoperative PTA (dB)	Postoperative WRS (%)	Mean FU Period (Months)	Other Hearing Outcome
Sudhoff et al. (2021) [13]	RS	7	7	No	?	?	?	?	13.4	SDS at 65 dB
Bento et al. (2016) [8]	CR	1	1	No	99	?	23	100	60	?
Pastor Gomis et al. (2021) [9]	CS	3	2	No	pz 1: L cophosis pz 2: R severe HL pz 3: L profoundHL	pz 3: SRS 20% at 60 dB SPL	pz 1: 25 pz 2: 30 pz 3: 40	?	pz 1: 36 pz 2: 24 pz 3: 24	SRS: pz 1: 90% at 65 dB SPL pz 2: 100% at 65 dB SPL
Häussler et al. (2021) [14]	CS	10	10	No	profound HL	?	?	?	pz 1: 6 pz 2: 12 pz 3: 12 pz 4: 24 pz 5: 0 pz 6: 24 pz 7: 0 pz 8: 6 pz 9: 6 pz 10: 1	FMT preCI (%): 0 FMT post CI (%): pz 1: 5 pz 2: 45 pz 3: 50 pz 4: 0 pz 5: 0 pz 6: 50 pz 7: 0 pz 8: 50 pz 9: 45 pz 10: 10
Carlson et al. (2016) [15]	RS	9 (10 ears)	10	7 (pz 4-10)	pz 1: 125 pz 2: 75 pz 3: 90 pz 4: 125 pz 5: 125 pz 6: 85 pz 7: 125 pz 8: 110 pz 9: 0 pz 10: 105	pz 1: 0 pz 2: 0 pz 3: 15 pz 4: 0 pz 5: 0 pz 6: 65 pz 7: 0 pz 8: 0 pz 9: 0 pz 10: 0	pz 1: 23 pz 2: 28 pz 3: 27 pz 4: ? pz 5: 27 pz 6: 25 pz 7: 28 pz 8: 22 pz 9: ? pz 10: 18	?	pz 1: 13 pz 2: 8 pz 3: 3 pz 4: 38 pz 5: 23 pz 6: 2 pz 7: 25 pz 8: 4 pz 9: 3 pz 10: 2	open-set word recognition score: -CNC median 50% [range, 28–88%] -AzBio median 73% [range, 60–91%]
Plontke et al. (2020) [16]	CR	1	1	No	?	?	?	75% at 65dB SPL	18	?
Plontke et al. (2018) [17]	CS	10	10	No	pz 1: 109 pz 2: >110 pz 3: >110 pz 4: >110 pz 5: 79 pz 6: >110 pz 7: 74 pz 8: 98 pz 9: >110 pz 10: 80	pz 1: 0 pz 2: 0 pz 3: 0 pz 4: 15 pz 5: 30 pz 6: 0 pz 7: 15 pz 8: 0 pz 9: 0 pz 10: 0	?	pz 1: 95 pz 2: ? pz 3: ? pz 4: ? pz 5: 70 pz 6: 30 pz 7: 80 pz 8: 95 pz 9: 65 pz 10: 75	pz 1: 24 pz 2: ? pz 3: ? pz 4: ? pz 5: 6 pz 6: 6 pz 7: 6 pz 8: 6 pz 9: 6 pz 10: 3	?
De Paula Vernetta et al. (2017) [10]	CR	1	1	No	89 dB	?	25	?	24	SRS 50% at 95 dB preop SRS 90% at 65 dB SPL postop
Kronenberg et al. (1999) [18]	CR	1	1	No	?	?	?	100	36	?
Ma et al. (2020) [19]	CR	1	1	No	profound HL	20	?	?	5	Speech reception testing 66% on BKB sentences and 79% on CUNY sentences.
Patel et al. (2022) [11]	CS	2	2	No	pz 1: severe HL pz 2: severe HL	pz 1: 0 pz 2: 0	pz 1: 37 pz 2: 25	pz 1: ? pz 2: 59	?	pz 1: 1 Mo pz 2: 6 Mo
Aschendorff et al. (2017) [20]	RS	8	5 (pz 2-4-5-6-8)	No	?	?	?	?	?	?
Plontke et al. (2017) [21]	CS	12	7	No	pz 1: 71 pz 2: >110 pz 3: 108.75 pz 8: 55 pz 11: 111.25	pz 1: 0 pz 2: 0 pz 3: 0 pz 8: 5 pz 11: 0	?	pz 1: 55 pz 2: 45 pz 3: 90 pz 8: 25 pz 11: 0	?	?
Plontke at al. (2020) [22]	CS	4	4	No	pz 1: >110 pz 2: 71 pz 3: >110 pz 4: 109	pz 1: 0 pz 2: 0 pz 3: 0 pz 4: 0	pz 1: 36 pz 2: 28 pz 3: 41 pz 4: 35	pz 1: 100 pz 2: 90 pz 3: 100 pz 4: 100	pz 1: 65 pz 2: 80 pz 3: 70 pz 4: 40	?
Totten et al. (2021) [12]	RS	20	14	No	pz 5: ° anacusia pz 8: ° anacusia	pz 5: ° ? pz 8: ° ?	pz 5: ° ? pz 8: ° ?	pz 5: ° ? pz 8: ° ?	?	?
Our experience	CS	2	2	1 (pz 1)	pz 1: R anacusia pz 2: 91.25	pz 1: R anacusia pz 2: ?	pz 1: 52,5 pz 2: 42,5	pz 1: SRS: 20% at 50 dB nHL pz 2: SDS 40 dB	pz 1: 26 pz 2: 3	pz 1: last FU: SDS 20 dB, SRS 90% at 70 dB nHL pz 2 last FU: PTA 35, SDS 50 dB, SRS 10% at 70 dB

* Regardless of the intravestibular involvement. Question marks (?) indicate unclear or no information provided. Abbreviations: ILS intralabyrinthine schwannomas, NF2 neurofibromatosis type 2, RS retrospective study, CR case report, CS case series, R right, L left, SRS speech recognition score, SDS speech detection score, HL hearing loss, FMT Freiburg Monosyllabic Test at 65 dB, CNC Consonant-Nucleus-Consonant, AzBio AzBio sentence test, BKB Bamford-Kowal-Bench sentence list, CUNY City University of New York sentences. ° received CI.

## Data Availability

Not applicable.
